# The Broad-Spectrum Antiviral Potential of the Amphibian Peptide AR-23

**DOI:** 10.3390/ijms23020883

**Published:** 2022-01-14

**Authors:** Annalisa Chianese, Carla Zannella, Alessandra Monti, Anna De Filippis, Nunzianna Doti, Gianluigi Franci, Massimiliano Galdiero

**Affiliations:** 1 Department of Experimental Medicine, University of Campania “Luigi Vanvitelli”, 80138 Naples, Italy; annalisa.chianese@unicampania.it (A.C.); carla.zannella@unicampania.it (C.Z.); anna.defilippis@unicampania.it (A.D.F.); massimiliano.galdiero@unicampania.it (M.G.); 2 Institute of Biostructures and Bioimaging (IBB), National Research Council (CNR), 80134 Naples, Italy; alessandra.monti@ibb.cnr.it (A.M.); nunzianna.doti@cnr.it (N.D.); 3 Department of Medicine, Surgery and Dentistry “Scuola Medica Salernitana”, University of Salerno, 84081 Baronissi, Italy

**Keywords:** antimicrobial peptides, SARS-CoV-2, HSV-1, antiviral drugs, coronavirus, antiviral therapy

## Abstract

Viral infections represent a serious threat to the world population and are becoming more frequent. The search and identification of broad-spectrum antiviral molecules is necessary to ensure new therapeutic options, since there is a limited availability of effective antiviral drugs able to eradicate viral infections, and consequently due to the increase of strains that are resistant to the most used drugs. Recently, several studies on antimicrobial peptides identified them as promising antiviral agents. In detail, amphibian skin secretions serve as a rich source of natural antimicrobial peptides. Their antibacterial and antifungal activities have been widely reported, but their exploitation as potential antiviral agents have yet to be fully investigated. In the present study, the antiviral activity of the peptide derived from the secretion of *Rana tagoi*, named AR-23, was evaluated against both DNA and RNA viruses, with or without envelope. Different assays were performed to identify in which step of the infectious cycle the peptide could act. AR-23 exhibited a greater inhibitory activity in the early stages of infection against both DNA (HSV-1) and RNA (MeV, HPIV-2, HCoV-229E, and SARS-CoV-2) enveloped viruses and, on the contrary, it was inactive against naked viruses (PV-1). Altogether, the results indicated AR-23 as a peptide with potential therapeutic effects against a wide variety of human viruses.

## 1. Introduction

Currently viral infections are reporting a dramatic increase in morbidity and mortality around the world. Despite the advent of vaccines able to prevent many of the previously fatal viral diseases, a wide variety of viruses still represent a serious threat for humanity. Since December 2019, the world population has been facing a novel and unrestrainable viral infection: several cases of pneumonia of unknown etiology have been reported in the city of Wuhan, China. Subsequently, the Chinese Centers for Disease Control and Prevention (CDC) recognized it as the Coronavirus disease 2019 (COVID-19), caused by the severe acute respiratory syndrome coronavirus 2 (SARS-CoV-2). On 11 March 2020, the World Health Organization (WHO) declared the beginning of the pandemic state, following the rapid spread of the virus and the high increase in infections that, to date, affected 265 million people worldwide [1,2,3]. Respiratory tract infections can be caused by other viruses, such as influenza and parainfluenza viruses, responsible for the frequent annual outbreaks with high morbidity rates as reported by the WHO and CDC: about 290,000–650,000 fatal cases per year have been recorded [4,5,6].

Despite considerable progress in the development of new antiviral therapies, results are not completely satisfying, and a lot remains to be completed [7,8]. The emergence of new drug-resistant strains and the absence of broad-spectrum antivirals represent some of the limits to be addressed [4,9,10].

Recently, antimicrobial peptides (AMPs), or host defense peptides, have attracted attention [4]. The AMPs are molecules present in the innate immune system of almost all living organisms, invertebrates, and vertebrates, identified as potential agents with therapeutic potential as they exhibit marked antibacterial, antiviral, antiparasitic and antifungal properties. AMPs are natural products derived from various microorganisms, including plants, arthropods, reptiles, and amphibians [8,11,12,13,14,15].

The AMPs derived from frog skin secretions represent a heterogeneous class which shares several characteristics: (i) the presence of basic amino acids conferring a net positive charge; (ii) the presence in the sequence of 50% hydrophobic amino acids; and (iii) the ability to assume an alpha-helix or a beta-sheet conformation during the interaction with the target cell membrane. As reported by Ladram et al., there are more than 40 families of AMPs deriving from anurans: the most known are represented by members belonging to the Pipidae family consisting of 5 genera (*Hymenochirus*, *Pseudhymenochirus*, *Pipa*, *Silurana*, and *Xenopus*) distributed in Africa and South America [16]. Among them, the magainins belonging to the genus *Xenopus laevis* showed high antiviral properties against Herpes simplex virus type-1 (HSV-1) and type-2 (HSV-2) [17], anticancer abilities against human small-cell lung cancer and bladder cancer cells [18,19], and additionally, antibacterial activities against Gram-positive and Gram-negative bacteria [20]. The antiviral activity of caerin 1.1, caerin 1.9, and maculatin 1.1 (produced by members of the Pelodryadidae family) has been described against human immunodeficiency virus type-1 (HIV-1), indicating a strong inhibitory action in the early stages of intracellular infection. Both caerin 1.1 (derived from the frog *Litoria caerula*) and caerin 1.9 (*Litoria chloris*) blocked the viral infection with an inhibition rate of 90% at 10 µM and > 99% at 5 µM, respectively. Furthermore, maculatin 1.1 (*Litoria genimaculata*) had an antiviral ability at 10 µM with 80% inhibition [21]. Furthermore, the dermoseptins, which are polycationic alpha-helix peptides derived from the Hylidae family of the genus *Phyllomedusa*, exhibited high antimicrobial activity. They interfered with both Gram-negative and Gram-positive bacteria, such as *Escherichia coli* (*E. coli*), with a minimum inhibitory concentration (MIC) at 1.5 µM, *Pseudomonas aeruginosa* (*P. aeruginosa*) (MIC 6.2 µM), *Staphylococcus aureus* (*S. aureus*) (MIC 25 µM), and against *Candida albicans* (*C. albicans*) (10 µM). Dermoseptins reduced infection of several viruses, such as HSV-1, HSV-2, HIV-1, and rabies virus, by acting in the early stage of the viral life cycle [22]. The Ranidae family, including *Rana temporaria*, *Rana tagoi*, *Rana muscosa*, and *Rana sakuraii*, constitutes the main source of amphibian AMPs. Temporins, derived from *Rana temporaria*, are peptides consisting of 10-13 amino acid residues with a slight cationic charge. Both temporins B, G, and SHa exert a remarkable antibacterial, antifungal, and antiviral activity. In detail, their action is principally focused on enveloped viruses, including HSV-1 [23,24,25], influenza A, and parainfluenza viruses [4], but the mechanism of action by which temporins exert their antiviral activity is different: (i) some can act at the intracellular level by destroying the viral envelope and/or preventing the virus-membrane host cell fusion (influenza virus, HSV-1); or (ii) others can interfere in viral replication by preventing the release of new virions (parainfluenza viruses) [4,10].

In 2003, Conlon et al. identified a new peptide derived from Japanese Ranid frogs (*Rana tagoi*) named AR-23, due to it being a 23 amino acid residues peptide, whose name was derived from the initial and final letter of the amino acids [26]. AR-23 is characterized by an amphipathic alpha-helix structure, and it is a melittin-related peptide (MRP), differing from melittin by two of the three positively charged residues at the C-terminus end (i.d. KNR) [27,28]. Several evidence indicated that melittin exhibited multiple anticancer, anti-inflammatory, and antimicrobial properties [8]. In detail, melittin directly interfered with the viral or bacterial external membranes, causing several damages ranging from pore formation to complete membrane lysis [29]. Despite a relative cytotoxicity, melittin showed an inhibitory effect against parasites (*Trypanosoma cruzi*), Gram-positive and Gram-negative bacteria (*S. aureus* MRSA, *S. epidermidis*, *E. coli*, and *Klebsiella pneumoniae*), and against a wide range of enveloped viruses with both DNA (HSV-1) and RNA (influenza A virus, HIV, Respiratory syncytial virus, and Vesicular stomatitis virus) genomes [8,30,31,32,33,34]. Uddin et al. demonstrated a marked antiviral activity of melittin against non-enveloped viruses [31]. Alternatively, Zhang et al. demonstrated that AR-23 had a bactericidal activity against Gram-positive bacteria, while it also had a reduced efficacy against Gram-negative ones [28].

However, AR-23 antiviral potential has not been investigated. In the present study, we evaluated the antiviral activity of AR-23 against a wide range of viruses, demonstrating a significant reduction in the viral infection with an inhibitory activity of peptide directed to the early phases of cell invasion.

## 2. Results

### 2.1. Cytotoxicity evaluation

The first step of our analysis was devoted to distinguishing antiviral activity from cellular toxicity. Therefore, to exclude the possibility that AR-23 could be cytotoxic on Vero cell monolayer, different concentrations of peptide in the range from 100 to 0.39 µM were tested by MTT assays. First, Vero cells were plated in a 96-well plate. Next, AR-23 was added at different concentrations. After 24 h, the peptide was removed and MTT solution was added to the cell monolayer for 3 h. Finally, dimethyl sulfoxide (DMSO) was used to dissolve the purple formazan, and absorbance was measured at 570 nm. The percentage of cell viability was calculated as a comparison to the not-treated cell.

**Figure 1:**
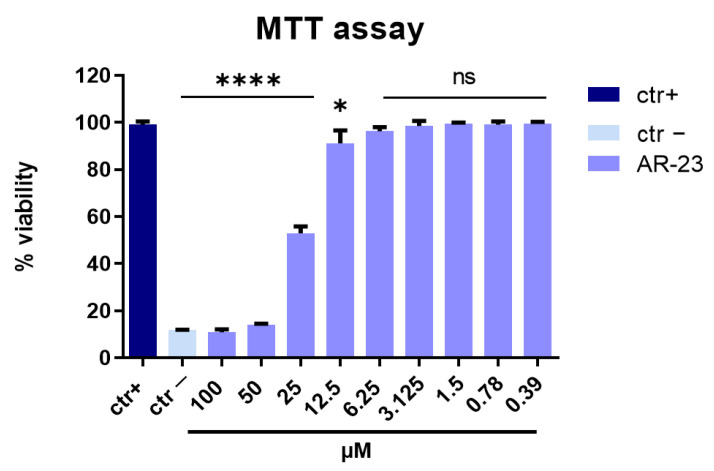
Cytotoxic activity. Cell viability evaluation by MTT assay of Vero cells after treatment with AR-23 for 24 h at several concentrations (from 100 to 0.39 μM). Not treated cells were used as positive control (ctr+), while DMSO was used as negative control (ctr-). **** *p* < 0.0001; * *p* < 0.0280; ns: non-significant.

As shown in Figure 1, AR-23 exhibited a relevant toxicity at the concentrations of 100 and 50 µM (>80%), and the 50% cytotoxic concentration (CC_50_) was at 25 µM. However, no toxicity or a negligible toxicity was detected in vitro following the peptide treatment in the range of concentration within 25 µM and 0.39 µM. Therefore, this range was used in all subsequent experiments. 

### 2.2. Evaluation of Antiviral Activity through Plaque Assay

In order to evaluate the antiviral activity and clarify the mechanism of action, AR-23 was tested against different types of viruses mainly responsible for human infections. Six antiviral experiments were performed at non-cytotoxic concentrations: (a) simultaneous addition of AR-23 and virus to the cells (co-treatment assay); (b) AR-23 incubated with the virus to test its antiviral activity (virus pre-treatment assay); (c) AR-23 incubated with the cells before the viral infection (cell pre-treatment assay); (d) AR-23 added to the cells already infected with virus (post-treatment assay); (e) cells infected with the virus at 4 °C and then treated with AR-23 at 37 °C (entry assay); and (f) AR-23 and virus incubated together with cells at 4 °C (attachment assay). The last two approaches (e, f) were performed only for HSV-1.

#### 2.2.1. Inhibitory Activity against DNA Virus: HSV-1

**Figure 2:**
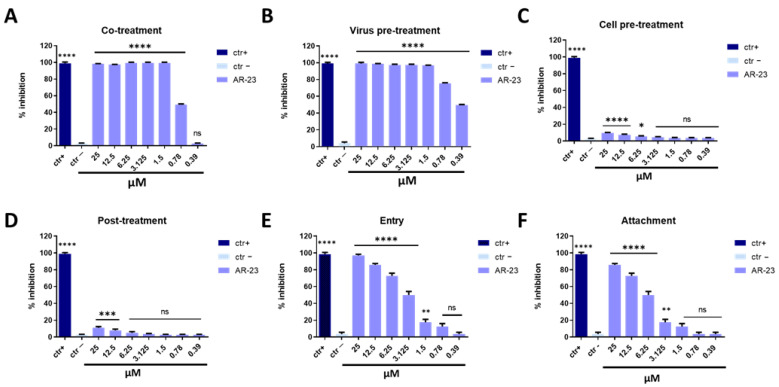
Antiviral activity against HSV-1. Different assays were performed in order to evaluate anti-HSV-1 activity. (**A**) Co-treatment: simultaneous addition of AR-23 and virus to the cells; (**B**) Virus pre-treatment: virus incubated with the peptide, and then used to infect cells; (**C**) Cell pre-treatment: AR-23 incubated with the cells before the viral infection; (**D**) Post-treatment: AR-23 added to the infected cells; (**E**) Entry: cells infected with the virus at 4 °C and then treated with AR-23 at 37 °C; (**F**) Attachment: AR-23 and virus incubated together with cells at 4 °C. The peptide inhibited the early stages of infection, acting in co-treatment (**A**) and virus pre-treatment (**B**) assays, while it was not able to interact with the cellular surface (**C**) or block the viral replication (**D**). AR-23 blocked the entry (**E**) and attachment (**F**) of HSV-1 on the host cell. Different compounds were used as positive control (ctr+) for each treatment: melittin (**A**,**B**, 5 μM for both the assays), dextran sulfate (**C**, 1 μM), aciclovir (**D**, 5 μM) [35], and heparin (**E**,**F**, 1 mg/mL for both the assays) [36], while infected cells were used as negative control (ctr-). **** *p* < 0.0001; *** *p* < 0.0002; ** *p* < 0.0021; * *p* < 0.021; ns: non-significant.

In the co-treatment assay (Figure 2A), HSV-1 and AR-23 were incubated together on the cell monolayer for 1 h at 37 °C. Then the mixture was removed and cell monolayer was washed and incubated with a viscous medium for 48 h. The results showed an evident reduction in the viral replication efficiency with half-maximal inhibitory concentration (IC_50_) of 0.78 μM. To investigate the mechanism of action of the peptide, additional experiments were carried out. Virus pre-treatment (Figure 2B) was a set experiment in which the peptide and virus were first incubated together at 37 °C for 1 h, and then the mixture was diluted with a medium and titrated on Vero cell monolayers. Only in this experiment, the multiplicity of infection (MOI) used was 0.1 for two reasons: (i) to reduce the concentration of the antiviral compound to one that was not active in an antiviral assay; and (ii) to allow the MOI to be 0.01 after dilution. Data showed consistently that with previous results, AR-23 interfered with the viral infection of the IC_50_ at 0.39 μM. Subsequently, Vero cells were pre-treated with AR-23 for 1 h before infection (cell pre-treatment assay, Figure 2C); alternatively, the cell monolayer was infected for 1 h and after-treated with peptide (post-treatment assay, Figure 2D). In both cases, AR-23 had a slight inhibitory effect, suggesting that the peptide was not able to stop infection by interacting with the cell membrane, and was not able to reach the cell internal compartment or to influence the virus replication once inside the cell. These data indicated that AR-23 could act in the extracellular phase of infection, most likely directly on the viral particles. The next question investigated if the peptide could act in the attachment and entry phase of HSV-1 infection. An attachment assay evaluated the ability of the peptide to interfere with the binding of the virus to the target cell. Vero cells were incubated together with peptide and virus at 4 °C to allow the viral attachment, but not the entry in the host cell. After 1 h, cells were washed and then incubated at 37 °C for 48 h. Alternatively, to evaluate the ability of the peptide to block the intracellular entry of HSV-1, an entry assay was performed. Here, cells were before-infected with the virus at 4 °C, and after-treated with AR-23. As represented in Figure 2E,F, AR-23 blocked the entry phase of the virus with an IC_50_ at 3.125 μM and consistently interfered with the viral attachment on the host cell membrane with an IC_50_ at 6.25 μM. For each treatment, the percentage of viral inhibition was evaluated by counting and comparing the plaques of AR-23-treated and infected cells to negative control (infected cells). Furthermore, several compounds were used as positive controls, i.e., melittin (for co-treatment and virus pre-treatment), dextran-sulfate (for cell pre-treatment), aciclovir (for post-treatment), and heparin (for entry and attachment assays).

**Figure 3:**
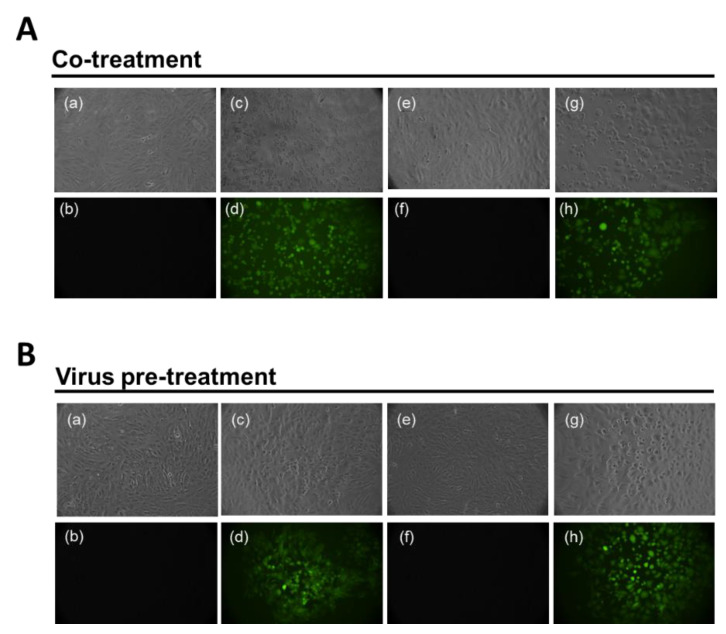
Antiviral activity against HSV-1-GFP. Evaluation of GFP expression in Vero cells infected by HSV-1 at 48 h post infection in co-treatment (**A**) and virus pre-treatment (**B**) assays. In the upper part of each panel, there are images visualized in RGB and, on the contrary, fuorescent images are reported below. (**a**,**b**) reported not infected and not treated cells; (**c**,**d**) indicated only infected cells; No plaques were present in (**e**,**f**) when cells were stimulated with AR-23 at 1.5 µM and 0.78 µM, in co-treatment (**A**) and virus pre-treatment (**B**); (**g**,**h**) reported plaques in cells treated with 0.39 µM of peptide in both the assays.

To validate our results, Vero cell monolayers were infected with an engineered HSV-1 in which the Green Fluorescent Protein (GFP) had been inserted into the genome sequence in the gene encoding VP22 tegument protein [37]. Co-treatment (Figure 3A) and virus pre-treatment (Figure 3B) assays were performed as described above, and fluorescence was recorded at 48 h post-infection. In the upper part of each panel, there are images visualized in Brightfield (RGB) and, on the contrary, fuorescent images are reported below. Data indicated that no fluorescence was observed at 1.5 μM (Figure 3A, **e** and **f**) when peptide and virus were incubated together on the cells; on the contrary, the not-active concentration of 0.39 μM (Figure 3A, **g** and **h**) caused a high fluorescence signal comparable to that of virus alone (Figure 3A, **c** and **d**). When the virus was placed in contact with peptide for 1 h and then used to infect cells, the GFP signal was absent also at the lower concentration of 0.78 μM (Figure 3B, **e** and **f**), meanwhile an intense fluorescence was present when cells were treated with 0.39 μM of AR-23 (Figure 3B, **g** and **h**). These data were in accordance with the plaque assay experiments.

#### 2.2.2. Inhibitory Activity against RNA Virus:

The antiviral effect of AR-23 was evaluated against enveloped and not enveloped RNA viruses, in detail against *Paramyxoviridae* (human parainfluenza virus type-2 and measles virus), *Coronaviridae* (HCoV-229E and SARS-CoV-2) and *Picornaviridae* (poliovirus 1) members.

##### *Paramyxoviridae* 

**Figure 4:**
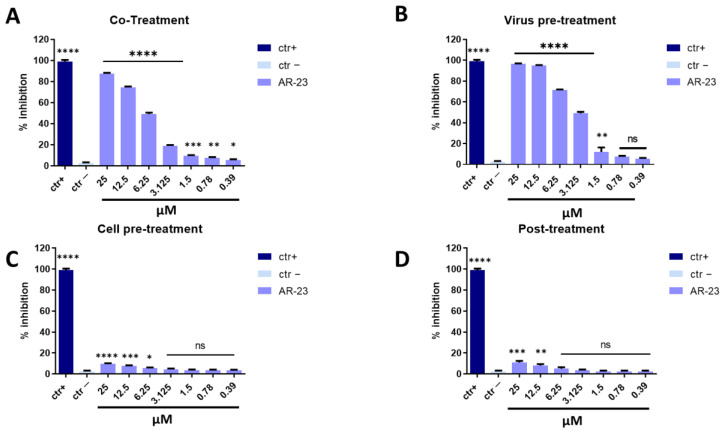
Antiviral activity against MeV. Different assays were performed in order to evaluate anti-MeV activity. (**A**) Co-treatment: simultaneous addition of AR-23 and virus to the cells; (**B**) Virus pre-treatment: virus incubated with the peptide, and then used to infect cells; (**C**) Cell pre-treatment: AR-23 incubated with the cells before the viral infection; (**D**) Post-treatment: AR-23 added to the infected cells. Peptide AR-23 inhibited the early stages of infection, acting in co-treatment (**A**) and virus pre-treatment (**B**) assays, while it was not able to interact with the cellular surface (**C**) or block the viral replication (**D**). Different compounds were used as positive control (ctr+) for each treatment: mucroporin M1 (**A**,**B**, 10 μM for both the assays) [40], FIP peptide (C, 20 μM) [41], and 5′-nor carbocyclic adenosine analogue (D, 5 μM) [42], while infected cells were used as negative control (ctr-). **** *p* < 0.0001; *** *p* < 0.0002; ** *p* < 0.0021; * *p* < 0.021; ns: non-significant.

**Figure 5:**
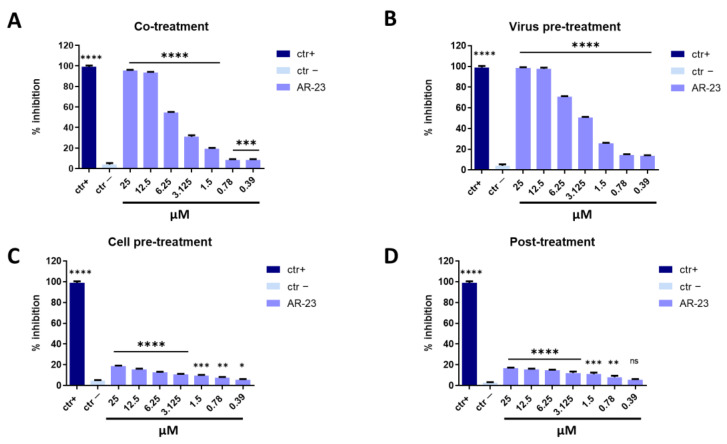
Antiviral activity against HPIV-2. Different assays were performed in order to evaluate anti-HPIV-2 activity. (**A**) Co-treatment: simultaneous addition of AR-23 and virus to the cells; (**B**) Virus pre-treatment: virus incubated with the peptide, and then used to infect cells; (**C**) Cell pre-treatment: AR-23 incubated with the cells before the viral infection; (**D**) Post-treatment: AR-23 added to the infected cells. Peptide AR-23 inhibited the early stages of infection, acting in co-treatment (**A**) and virus pre-treatment (**B**) assays, while it was not able to interact with the cellular surface (**C**) or block the viral replication (**D**). Different compounds were used as positive control (ctr+) for each treatment: mucroporin M1 (**A**,**B**, 10 μM for both the assays) [40], FIP peptide (C, 20 μM) [41], and 5′-nor carbocyclic adenosine analogue (D, 5 μM) [42], while infected cells were used as negative control (ctr-). **** *p* < 0.0001; *** *p* < 0.0002; ** *p* < 0.0021; * *p* < 0.021; ns: non-significant.

Measles virus (MeV) and Human parainfluenza virus type-2 (HPIV-2) are enveloped viruses belonging to the *Paramyoviridae* family with a single-stranded, negative-sense RNA. These viruses are highly contagious and are transmitted via the respiratory route causing fever, skin rash (for MeV), alveolitis, and pneumonitis (for HPIV-2) [38,39]. The same conditions described above were set to identify the step in viral life cycle that could be inhibited by the peptide (co-treatment, virus pre-treatment, cell pre-treatment, and post-treatment assays). In all conditions tested for MeV and HPIV-2, the inhibitory activity trends were very similar to what was observed against HSV-1, but a lower antiviral effect was recorded (Figure 4 and Figure 5). AR-23 showed high inhibitory activity when added simultaneously to the virus and cells in co-treatment assays, or when it was incubated for 1 h with virus and then added on the cells in virus pre-treatment assays, evidencing that AR-23 is endowed with a strong inhibitory activity directed toward the viral envelopes. However, no antiviral activity was observed in the other two assays, i.e., cell pre-treatment and post-treatment, indicating that AR-23 did not interact with the cell surface or interfere in viral replication. Setting the threshold line at IC_50_, AR-23 showed a remarkable antiviral potential at 6.25 μM against MeV and HPIV-2 in co-treatment test, while IC_50_ was 3.125 μM in virus pre-treatment assay for both paramyxoviruses.

##### *Coronaviridae* 

**Figure 6:**
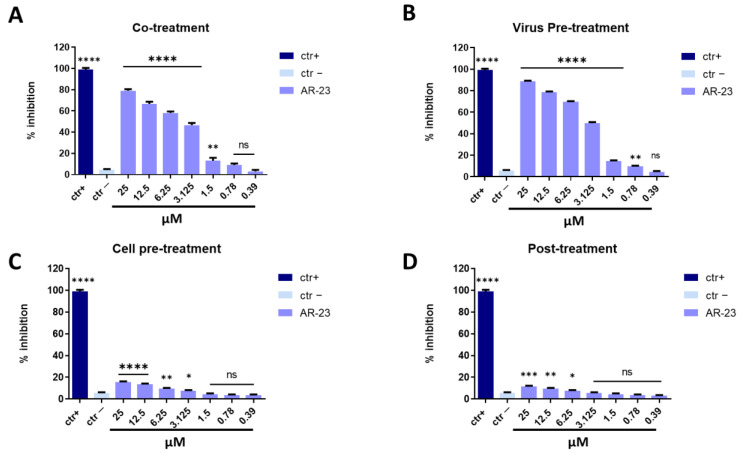
Antiviral activity against alphacoronavirus (HCoV-229E). Different assays were performed in order to evaluate anti-HCoV-229E activity. (**A**) Co-treatment: simultaneous addition of AR-23 and virus to the cells; (**B**) Virus pre-treatment: virus incubated with the peptide, and then used to infect cells; (**C**) Cell pre-treatment: AR-23 incubated with the cells before the viral infection; (**D**) Post-treatment: AR-23 added to the infected cells. Peptide AR-23 inhibited the early stages of infection, acting in co-treatment (**A**) and virus pre-treatment (**B**) assays, while it was not able to interact with the cellular surface (**C**) or block the viral replication (**D**). Different compounds were used as positive control (ctr+) for each treatment: rhamnolipids M15RL (**A**,**B**, 50 μg/mL for both the assays) [43,44], ivermectin (**C**, 10 μM) [45], and remdesivir (**D**, 10 μM) [45], while infected cells were used as negative control (ctr-). **** *p* < 0.0001; *** *p* < 0.0002; ** *p* < 0.0021; * *p* < 0.021; ns: non-significant.

**Figure 7:**
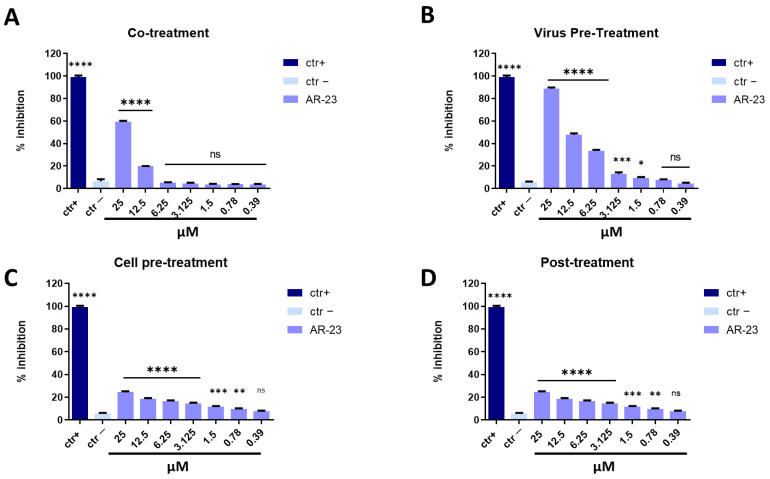
Antiviral activity against betacoronavirus (SARS-CoV-2). Different assays were performed in order to evaluate anti-SARS-CoV-2 activity. (**A**) Co-treatment: simultaneous addition of AR-23 and virus to the cells; (**B**) Virus pre-treatment: virus incubated with the peptide, and then used to infect cells; (**C**) Cell pre-treatment: AR-23 incubated with the cells before the viral infection; (**D**) Post-treatment: AR-23 added to the infected cells. Peptide AR-23 inhibited the early stages of infection, acting in co-treatment (**A**) and virus pre-treatment (**B**) assays, while it was not able to interact with the cellular surface (**C**) or block the viral replication (**D**). Different compounds were used as positive control (ctr+) for each treatment: rhamnolipids M15RL (**A**,**B**, 50 μg/mL for both the assays) [43], ivermectin (**C**, 10 μM) [45], and remdesivir (**D**, 10 μM) [45], while infected cells were used as negative control (ctr-). **** *p* < 0.0001; *** *p* < 0.0002; ** *p* < 0.0021; * *p* < 0.021; ns: non-significant.

To investigate whether AR-23 could exhibit similar activity against other enveloped RNA viruses, the antiviral potential was analyzed against HCoV-229E and SARS-CoV-2, belonging to the *Coronaviridae* family. As observed for paramyxoviruses, AR-23 showed a strong activity against *Coronaviridae* members (Figure 6 and Figure 7). In agreement with previous data, AR-23 was more efficacious in virus pre-treatment and co-exposure experiments against the alphacoronavirus HCoV-229E, by showing the 50% inhibition at 3.125 μM.

AR-23 showed 60% of inhibition at 25 μM in co-treatment experiments and 50% at 12.5 μM in virus pre-treatment assay against SARS-CoV-2, while it was inactive in cell pre-treatment and post-treatment conditions (Figure 7). Overall, these results suggested that the antiviral activity of AR-23 is generally directed toward the early stages of viral entry.

##### *Picornaviridae* 

**Figure 8:**
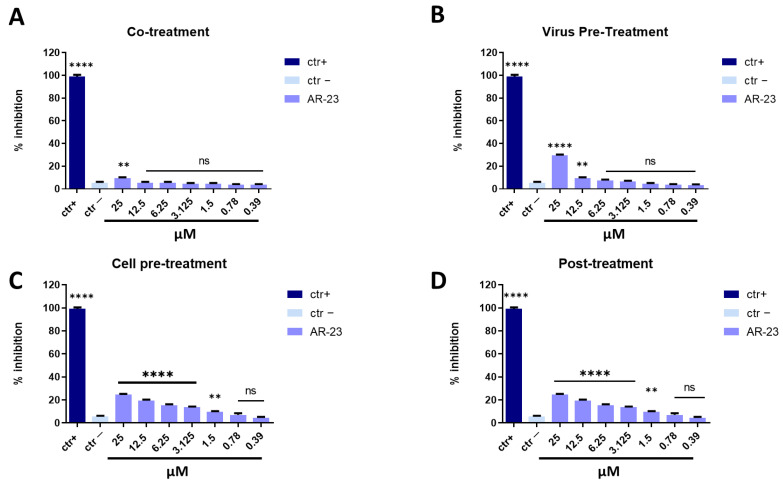
Antiviral activity against PV-1. Different assays were performed in order to evaluate anti-PV-1 activity. (**A**) Co-treatment: simultaneous addition of AR-23 and virus to the cells; (**B**) Virus pre-treatment: virus incubated with the peptide, and then used to infect cells; (**C**) Cell pre-treatment: AR-23 incubated with the cells before the viral infection; (**D**) Post-treatment: AR-23 added to the infected cells. Peptide AR-23 inhibited the early stages of infection, acting in co-treatment (**A**) and virus pre-treatment (**B**) assays, while it was not able to interact with the cellular surface (**C**) or block the viral replication (**D**). Different compounds were used as positive control (ctr+) for each treatment: pleconaril (**A**,**B**, 2 μg/mL for both the assays) [46], WIN51711 (**C**, 5 μg/mL) [45], and protein 2C (D, 10 μM) [47], while infected cells were used as negative control (ctr-). **** *p* < 0.0001; ** *p* < 0.0021; * *p* < 0.021; ns: non-significant.

Poliovirus type 1 (PV-1), an enterovirus belonging to the *Picornaviridae* family, was used as a viral model without envelope with a single-stranded, positive-sense RNA genome. The antiviral activity of AR-23 against PV-1 was assayed in the same conditions used for other viruses previously described. As reported in Figure 8, AR-23 was not able to show any relevant activity against PV-1. Our conclusions are that the main target for AR-23 is the lipidic membrane of enveloped viruses.

### 2.3. Molecular Assay: Real-Time PCR

**Figure 9:**
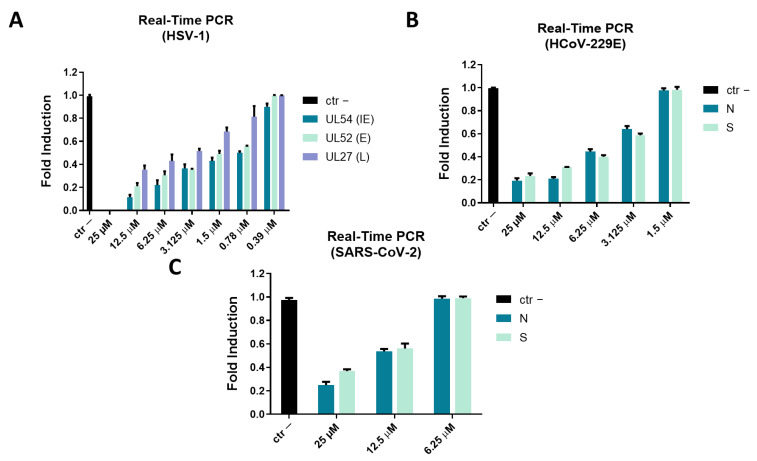
Molecular assay. Real-time PCR was performed to evaluate the effect of AR-23 on viral gene expression. Virus pre-treatment assay was performed, RNA was extracted after 24 h, retro-transcribed into cDNA and amplified by real-time PCR. The expression of UL54, UL52, and UL27 genes was analyzed for HSV-1 (**A**). The expression of N and S was evaluated for HCoV-229E (**B**) and SARS-CoV-2 (**C**). Ctr- refers to infected but not treated cells.

To confirm data obtained in cell culture through plaque assays, a molecular test was carried out. Expression of genes involved in the viral infection was analyzed, in detail UL54, UL27, and UL52 genes for DNA virus (HSV-1), and Spike gene (S) and Nucleocapsid gene (N) for RNA viruses (HCoV-229E and SARS-CoV-2). In detail, UL54 is an immediate early gene encoding for the ICP27 protein involved in HSV-1 replication, by inhibiting mRNA splicing at the post-transcriptional level and promoting the export of viral transcripts; UL52 is an early gene codifying for DNA primase, and UL27 gene represents a late gene coding for the structural glycoprotein B (gB) [48]. Virus pre-treatment assay was performed, RNA was collected after 24 h, retro-transcribed into cDNA and amplified by real-time PCR. Data showed that AR-23 interfered with HSV-1 replication by significantly reducing the expression of all three genes (Figure 9A); by decreasing the peptide concentration, viral gene expression increased and reached the same level of virus control at the concentration of 0.39 μM. Antiviral activity of the peptide was investigated against HCoV-229E and SARS-CoV-2, analyzing the expression of proteins involved in the entry of the virus in the cell (S protein), and the viral genome packaging (N protein) [49]. Results showed that the infection was reduced in a dose-dependent manner for both tested viruses (Figure 9B,C).

### 2.4. Morphological Analysis of AR-23 by Transmission Electron Microscope (TEM)

**Figure 10:**
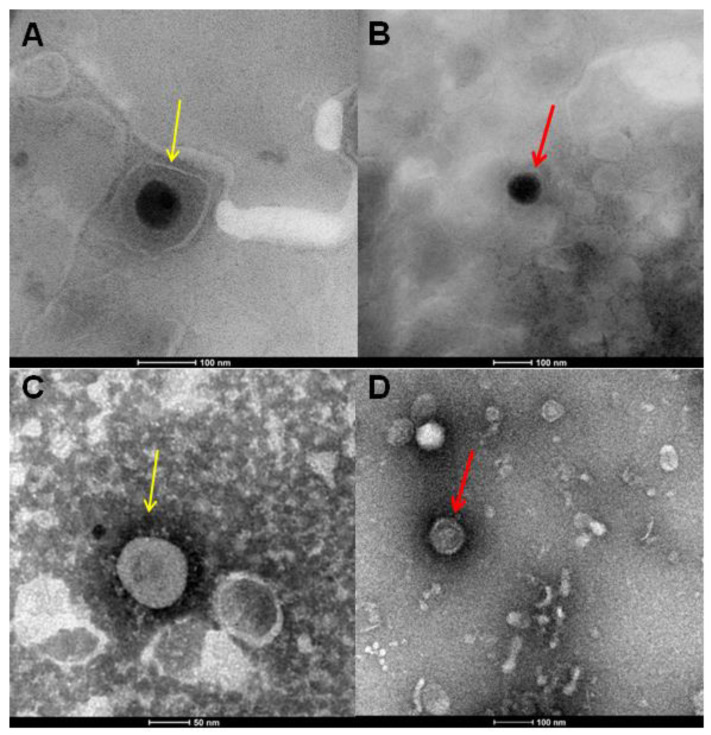
TEM analysis. TEM images of HSV-1 (**A**,**B**), as well as SARS-CoV-2 (**C**,**D**) virions. In (**A**), yellow arrows show the untreated HSV-1 particles. In (**C**), spike proteins of untreated SARS-CoV-2 virions are indicated by yellow arrows. In (**B**,**D**), red arrows show naked particles of HSV-1 and SARS-CoV-2, respectively, treated with 25 µM of AR-23.

Further evidence showed that AR-23 could act on the viral envelope by blocking the infection of different viruses that was obtained by treating HSV-1 and SARS-CoV-2 virions with the peptide and observing them by TEM (Figure 10). In detail, the purified virus was incubated with AR-23 at 25 µM, transferred to carbon-coated copper grids and negative staining was performed with 2% phosphotungstic acid (pH 6.5).

Figure 10A shows untreated HSV-1, where from the outside to the inside we can observe the envelope, the tegument, and the capsid. When the virus was treated with AR-23 at 25 μM (Figure 10B), the viral particles lose the envelope and, therefore, the ability to infect cells. Figure 10C shows SARS-CoV-2 virions with S proteins protruding from the viral surface. Treatment with AR-23 at 25 μM (Figure 10D) showed an effect very similar to what has already been observed with HSV-1: the peptide could act by detaching the S proteins from the envelope causing the viral inactivation. This evidence indicated that the peptide AR-23 could have a detergent-like action on the viral envelope, resulting in the loss of the infectious potential of the virus and, therefore. blocking its entire life cycle.

## 3. Discussion

The COVID-19 pandemic has garnered the attention of the world’s population towards emerging and reemerging viral infections. Since the rapid development of drug-resistant strains, viral diseases represent a major global health concern, and it is mandatory to identify new antiviral effective agents [10,28,50]. Antiviral peptides (AVPs) are a group of peptides representing an important armamentarium for the discovery of new therapies to combat viral infection. Several AVPs have been identified, in particular entry/fusion inhibitors, such as enfuvirtide (Fuzeon) for treatment of HIV-1 infection [51,52]. In the present study, we analyzed the antiviral activity of AR-23, the AMP derived from the skin secretions of the amphibian *Rana tagoi*, a melittin-related peptide. Although both peptides derive from different sources (*Rana tagoi* and *Apis millifera*, respectively), they share a high similarity in the amino acid sequence. Melittin is well-known for its broad-spectrum activity against bacteria, fungi, viruses, and for the anticancer properties. Alternatively, AR-23 reported significant bactericidal activity, but no studies described its antiviral potential. Zhang et al. evaluated the antibacterial activity of the peptide AR-23 [28] against both Gram-positive and Gram-negative bacteria, showing slightly lower inhibition of AR-23 when compared to melittin. Both melittin and AR-23 were active against *E. coli* (MIC 12.5 µM and 25 µM, respectively), *P. aeruginosa* (MIC 6.25 µM and 12.5 µM), *K. pneumoniae* (MIC 6.25 µM and 12.5 µM), *S. aureus* (MIC 3.13 µM and 6.25 µM), *S. epidermidis* (MIC 3.13 µM for both the peptides), and *Bacillus subtilis* (*B. subtilis*) (MIC 3.13 µM for both the peptides) [28]. Urban et al. demonstrated an effective antimicrobial activity of AR-23 against the clinical isolate of methicillin-resistant *S. aureus* (MRSA) [27]. While the antibacterial potential of AR-23 has been widely reported, its antiviral effect is still to be investigated. On the other side, it has already been demonstrated that melittin is able to interfere with a broad spectrum of viruses (HSV-1, HSV-2, Influenza virus, flaviviruses, and HIV-1) [8]. Our data indicated for the first time that AR-23 can inhibit the viral replication when pre-incubated with the virus, showing an IC_50_ at 0.39 µM for HSV-1, 3.125 µM for MeV, HPIV-2 and HCoV-229E, and 12.5 µM for SARS-CoV-2. Since the greatest antiviral activity was recorded against HSV-1, the antiviral activity of AR-23 has been studied in detail against this virus. We demonstrated that AR-23 affected the early stages of herpetic infection, i.d. the process of attachment and entry in the host cell (IC_50_ at 6.25 µM and 3.12 µM, respectively). The inhibition of HSV-1 infectivity was more limited than that recorded in virus pre-treatment assay, suggesting that the peptide could interact directly with the viral particles, and its mechanism of action could involve in part the blocking of the extracellular and early phases of infection. This action was shared for all the enveloped viruses and not for the naked viruses, such as the poliovirus, evidencing that AR-23 could act on the external viral layer, specifically on the envelope glycoproteins. These data were supported by TEM analysis. AR-23 has shown a detergent-like action, since the viral envelope resulted damaged, both in HSV-1 and SARS-CoV-2. Finally, the expression of viral genes was evaluated by RT-qPCR for each virus, confirming the data obtained in vitro by plaque assay. Altogether the results suggested that AR-23 could represent a new promising antiviral agent able for preventing or combating the main viral diseases widespread in the world population.

## 4. Materials and Methods

### 4.1. Peptide Synthesis and Characterization

Protected amino acids, coupling agents (HATU, Oxyma) and Fmoc-Rink Amide AM resin used for peptide synthesis, solvents, including acetonitrile (CH_3_CN), dimethylformamide (DMF) and other products such as trifluoroacetic acid (TFA), sym-collidine, diisopropylethylamine (DIPEA), and piperidine, were purchased from Merck (Milan, Italy). The peptide AR-23 (single letter sequence: H-AIGSILGALAKGLPTLISWIKNR-NH2) was synthesized using oxyma/DIC as coupling agents and following methods reported in the literature [53]. The HPLC preparative purification was carried out on a WATERS 2545 preparative system (Waters, Milan, Italy) fitted out with a WATERS 2489 UV/Visible detector, applying a linear gradient of CH_3_CN/0.05%TFA in water 0.05% TFA from 5 to 70% of in 20 min, at a flow rate of 12 mL/min. MS characterization of the peptide was performed using an ESI-TOF-MS Agilent 1290 Infinity LC System coupled to an Agilent 6230 time-of-flight (TOF) LC/MS System (Agilent Technologies, Cernusco sul Naviglio, Italy). The LC Agilent 1290 LC module was coupled with a photodiode array (PDA) detector and a 6230 time-of-flight MS detector, along with a binary solvent pump degasser, a column heater, and an autosampler. LC-MS characterization of the peptide was performed using a C18 Waters xBridge column (3 μm, 4.6 × 5.0 mm), applying a linear gradient of CH3CN/0.05% TFA in water 0.05% TFA from 5 to 70% of in 20 min, at a flow rate of 0.2 mL/min. The yields of the target peptides, calculated as ((experimental weight of pure peptide)/(theoretical weight) x 100), where the theoretical weight was calculated based on the synthesis scale used were estimated to be about 70%. The relative purity of peptide was calculated as the ratio of peak area of the target peptide and the sum of areas of all detected peaks from the UV chromatograms at 210,4 nm. The purity was >98%.

### 4.2. Cell Culture and Viral Strain

Vero cells (ATCC CCL-81, Manassas, Virginia, United States) and Vero/hSLAM cells (ECACC 04091501, Porton Down, United Kingdom) were grown in Dulbecco’s Modified Eagle Medium (DMEM) with 4.5 g/L glucose (Microtech, Naples, Italy) supplemented with antibiotic solution 100× (Himedia, Naples, Italy) and 10% Fetal Bovine Serum (Microtech).

HSV-1 (strain SC16), containing a lacZ gene driven by the cytomegalovirus IE-1 promoter to express beta-galactosidase, and fluorescent HSV-1, containing the GFP reporter inserted into the gene coding for the VP22 tegument protein [54], were propagated on Vero cells, as previously reported [37]. Measle virus (ATCC VR-24) was grown on VERO/hSLAM cells, while HPIV-2 (ATCC VR-92), HCoV-229E (ATCC VR-740), and SARS-CoV-2 (strain VR PV10734, kindly donated by the Lazzaro Spallanzani Hospital of Rome, Italy) and Enterovirus C (Sb-1, poliovirus Sabin strain chat, ATCC VR-1562) on Vero cell line.

### 4.3. Cytotoxic Activity

Vero cells were seeded in triplicate at 2 × 10^4^ cells/mL in a 96-well plate for 24 h. The next day, cells were incubated with several concentration of peptide (from 100 to 0.39 μM) and after 24 h all wells were treated with 100 μL 3-(4,5-Dimethylthiazol-2-yl)-2,5-Diphenyltetrazolium Bromide (MTT, Sigma-Aldrich, St. Louis, MO, US) solution (5 mg/mL) and incubated at 37 °C/5% CO_2_ for 3h; 100 μL DMSO (Sigma-Aldrich) was added to each well to dissolve the purple formazan, and absorbance was measured at 570 nm. A total of 100 μL DMSO was used for negative control (ctr-), while 100 μL of culture medium represented the positive control (ctr+).

### 4.4. Antiviral Activity

Vero cells or Vero/hSLAM were seeded (2 × 10^5^) in 24-well plates 24 h before use. Four treatment assays were performed:

(a) Co-treatment assay: cells were inoculated with several concentrations of peptide (from 100 to 0.78 μM) and virus at MOI 0.01 for 1 h at 37 °C/5% CO_2_.
(b) Virus pre-treatment assay: peptide was incubated together with virus at MOI 0.1, for 1 h at 37 °C. After that, the mixture was inoculated on the cells for 1 h at 37 °C/5% CO_2_.
(c) Cell pre-treatment assay: cells were first treated with peptide for 1 h, after that AR-23 was removed and cells were infected with virus (MOI 0.01) for another hour at 37 °C/5% CO_2_.
(d) Post-treatment assay: cells were infected with virus (MOI 0.01) for 1 h and, subsequently, peptide was added for another hour at 37 °C/5% CO_2_.

At the end, for each treatment cells were washed with citrate buffer (pH 3) and overlaid with DMEM supplemented with carboxymethylcellulose (CMC) 5% for 48–72 h. Then, 4% formaldehyde and 0.5% crystal violet were used to fixed and stained cells, respectively. Plaques were counted and the percentage of viral inhibition was calculated in relation to no treated control (ctr-), as followed:

% viral inhibition = (100 − (plaques counted in the test sample)/plaques counted in the negative control)) × 100.

Two additional assays were performed:

(e) Attachment assay: Vero cells were seeded in 24-well plates at the density of 2.5 × 10^5^ cells/mL for 24 h. Then, they were infected with HSV-1 (MOI 0.01) and at same time treated with AR-23 from 25 to 0.39 μM for 1 h at 4 °C. Subsequently, the cell monolayer was washed and incubated for 1 h at 37 °C with a culture medium. Then, cells were washed with PBS and DMEM/CMC was added for 48 h at 37 °C/5% CO_2_.
(f) Entry assay: Vero cells were plated in 24-well plates as indicated above and then infected with HSV-1 (MOI 0.01) for 1 h at 4 °C. Subsequently, AR-23 was put on cells for 1 h at 37 °C and finally cells were washed with citrate buffer (pH 3) and overlaid with DMEM/CMC.

As in the other antiviral assays, viral plaques were counted, and the percentage of infectivity inhibition was evaluated by counting and comparing the plaques of AR-23-treated and infected cells to negative control (infected cells).

### 4.5. Analysis of Gene Expression

Cells were seeded at the same conditions previously reported [55]. The next day, virus pre-treatment assay was performed, and after 48 h RNA was extracted by TRIzol reagent (Thermo Fisher Scientific, Waltham, MA, United States) and quantified by evaluating the absorbance at Nanodrop (NanoDrop 2000, Thermo Fisher Scientific). The 5× All-In-One RT MasterMix (Applied Biological Materials, Richmond, Canada) was used to retrotranscribe RNA to cDNA, and through a quantitative polymerase chain reaction, 2 µL of cDNA were amplified. The expression of the following viral genes was evaluated: UL54, UL52, and UL27 (for HSV-1), S and N proteins (for HCoV-229E and SARS-CoV-2). Relative target threshold cycle (Ct) values were normalized to Glyceraldehyde 3-phosphate dehydrogenase (GAPDH), used as housekeeping gene. The mRNA levels of cells treated with AR-23 were expressed using the 2-ΔΔCt method. In Table 1, the sequences of primers for the Real-time PCR are reported. Primers were provided by Eurofins (Ebersberg, Germany).

### 4.6. Virus Purification and TEM Analysis

The viruses were purified through density gradient ultracentrifugation with cesium chloride (CsCl) as previously reported [56]. Transmission electron microscopy (TEM) analysis was performed using the FEI TECNAI G2 S-twin electron microscope. The preparation of the samples was carried out by incubating for 1 h at 37 °C; a drop (5 µL) of the purified virus with a drop of AR-23 at 25 µM. Virus control was prepared by incubating, under the same conditions, 5 µL of the purified virus with 5 µL of culture medium. Subsequently, 5 µL of the suspension was transferred to carbon-coated copper grids (Merck, Readington Township, NJ, US) and allowed to evaporate overnight. Negative staining was performed by using 2% phosphotungstic acid (pH 6,5). A drop of dye (5 µL) was transferred to the grid for 30 s and the excess was removed with filter paper, improving the contrast.

### 4.7. Statistical Analysis

All tests were performed in triplicate and expressed as mean ± Standard Deviation (SD) calculated by GraphPad Prism, version 8.0.1; Software for 2D graphing and statistics; GraphPad Software Inc.: San Diego, CA, US, 2018. One-way ANOVA followed Dunnett’s multiple comparisons test was performed; a value of *p* ≤ 0.05 was considered significant.

## 5. Conclusions

In conclusion, we analysed for the first time the antiviral potential of the frog skin derived peptide AR-23. It exhibited a broad-spectrum activity against all enveloped viruses, such as herpesvirus, paramyxoviruses, and coronaviruses, including SARS-CoV-2. We hypothesized that AR-23 principal targets could be the viral surface, and, at the same time, its action could be also ascribed to a damage on the viral envelope.

## Figures and Tables

**Table 1:** Primers for viral genes used in Real-time PCR.

Gene Symbol	Forward Sequence	Reverse Sequence
HSV-1 UL54	TGGCGGACATTAAGGACATTG	TGGCCGTCAACTCGCAG
HSV-1 UL52	GACCGACGGGTGCGTTATT	GAAGGAGTCGCCATTTAGCC
HSV-1 UL27	GCCTTCTTCGCCTTTCGC	CGCTCGTGCCCTTCTTCTT
HCoV-229E S	CGTTGAACTTCAAACCTCAGA	ACCAACATTGGCATAAACAG
HCoV-229E N	GTCGTCAGGGGTAGAATACCTTA	CCCGTTTGCCCTTTCTAGT
SARS-CoV-2 S	AGGTTGATCACAGGCAGACT	GCTGACTGAGGGAAGGAC
SARS-CoV-2 N	GGGGAACTTCTCCTGCTAGAAT	CAGACATTTTGCTCTCAAGCTG
GAPDH	CCTTTCATTGAGCTCCAT	CGTACATGGGAGCGTC

## Data Availability

The data presented in this study are available on request from the corresponding author. Authors can confirm that all relevant data are included in the article.
